# Metformin reduces saturated fatty acid-induced lipid accumulation and inflammatory response by restoration of autophagic flux in endothelial cells

**DOI:** 10.1038/s41598-020-70347-w

**Published:** 2020-08-11

**Authors:** Hae-Suk Kim, Guang Ren, Teayoun Kim, Sushant Bhatnagar, Qinglin Yang, Young Yil Bahk, Jeong-a Kim

**Affiliations:** 1 Department of Medicine, Division of Endocrinology, Diabetes, and Metabolism, Comprehensive Diabetes Center, University of Alabama at Birmingham, 1825 University Blvd, Birmingham, AL 35294 USA; 2 Department of Nutrition, University of Alabama at Birmingham, Birmingham, AL USA; 3 Department of Biotechnology, College of Biomedical and Health Science, Konkuk University, Chungju, 27478 Republic of Korea

**Keywords:** Macroautophagy, Vascular diseases, Biochemistry

## Abstract

Autophagy, an integral part of the waste recycling process, plays an important role in cellular physiology and pathophysiology. Impaired autophagic flux causes ectopic lipid deposition, which is defined as the accumulation of lipids in non-adipose tissue. Ectopic lipid accumulation is observed in patients with cardiometabolic syndrome, including obesity, diabetes, insulin resistance, and cardiovascular complications. Metformin is the first line of treatment for type 2 diabetes, and one of the underlying mechanisms for the anti-diabetic effect of metformin is mediated by the stimulation of AMP-activated protein kinase (AMPK). Because the activation of AMPK is crucial for the initiation of autophagy, we hypothesize that metformin reduces the accumulation of lipid droplets by increasing autophagic flux in vascular endothelial cells. Incubation of vascular endothelial cells with saturated fatty acid (SFA) increased the accumulation of lipid droplets and impaired autophagic flux. We observed that the accumulation of lipid droplets was reduced, and the autophagic flux was enhanced by treatment with metformin. The knock-down of AMPKα by using siRNA blunted the effect of metformin. Furthermore, treatment with SFA or inhibition of autophagy increased leukocyte adhesion, whereas treatment with metformin decreased the SFA-induced leukocyte adhesion. The results suggest a novel mechanism by which metformin protects vascular endothelium from SFA-induced ectopic lipid accumulation and pro-inflammatory responses. In conclusion, improving autophagic flux may be a therapeutic strategy to protect endothelial function from dyslipidemia and diabetic complications.

## Introduction

Autophagy is a lysosomal catabolic process that degrades misfolded proteins, accumulated lipids, and damaged mitochondria^1–5^. Macroautophagy (autophagy hereafter) involves autophagosome formation (double membranous structure in the cytoplasm) and subsequent fusion with lysosome followed by degradation of the sequestered materials in autolysosome by lysosomal hydrolases. This lysosomal catabolic process is crucial for cellular homeostasis, differentiation, survival, immune response, and development in metazoans^6,7^. Dysregulated autophagy contributes to aging, cancer, infections, neurological disorders, and cardiometabolic diseases^8–11^. Thus, maintaining normal autophagy is essential for a healthy physiology.

One of the parameters indicating cellular homeostasis is autophagic flux^12,13^, which is a balance between autophagosome biogenesis (sequestration of multiple autophagic proteins and formation of membrane-like structure) and degradation of enclosed materials. Suppressed autophagic flux results in the accumulation of un-necessary cargoes, which contributes to endoplasmic reticulum (ER) stress, mitochondrial dysfunction, and ectopic lipid accumulation^14–19^. Conversely, the stimulation of autophagic flux improves both metabolic and cardiovascular functions^20–22^. Thus, there may be a link between impaired autophagic flux and cardiometabolic disease.

Circulating fatty acid levels are elevated in subjects with obesity, insulin resistance, dyslipidemia, and diabetes^23,24^. The effects of saturated fatty acid (SFA) on endothelial cells contribute to insulin resistance and endothelial dysfunction^25–27^. We previously reported that SFA stimulates pro-inflammatory responses and endoplasmic reticulum (ER) stress in vascular endothelial cells through toll-like receptor-mediated mechanisms^25,26^. This suggests that the SFA-induced pro-inflammatory response in endothelial cells plays an important role in both metabolism and cardiovascular functions.

Metformin is the most widely prescribed drug for patients with type 2 Diabetes Mellitus (T2DM), and it is a known stimulator of AMP-activated protein kinase (AMPK)^28^. Activation of AMPK by metformin suppresses hepatic glucose production, increases glucose uptake in the skeletal muscle, and stimulates vasodilation by activation of eNOS^28–31^. AMPK is involved in the initiation of autophagy by directly phosphorylating Unc51-like kinase 1 (ULK1), which recruits proteins required for autophagosome formation^32^. However, the effects of metformin on lipid accumulation and pro-inflammatory response in vascular endothelial cells are unknown. In the present study, we demonstrate the effect of metformin in SFA-induced impairment of autophagic flux and its roles in ectopic lipid accumulation and pro-inflammatory responses in vascular endothelial cells.

## Results

### Saturated fatty acids, but not unsaturated fatty acids affect autophagy

**Figure 1:**
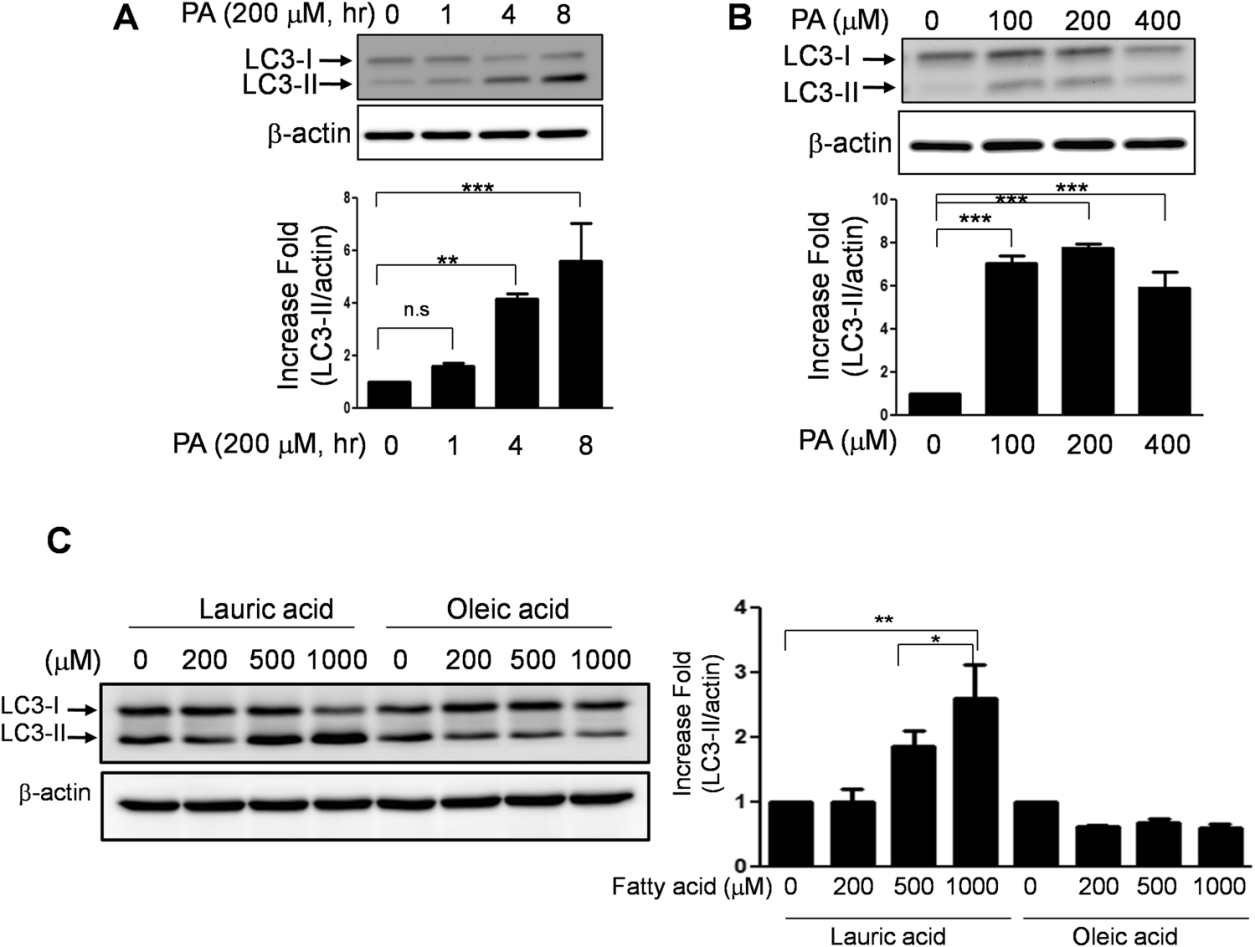
Saturated fatty acids, but not unsaturated fatty acids, affect autophagy. Human endothelial cells were serum starved for 2 h, and then (**A**) treated with PA (200 μM) for the indicated durations; (**B**) treated with BSA, or PA (indicated concentrations) for 4 h; (**C**) treated with lauric acid or oleic acid at the indicated concentrations for 4 h. The cell lysates were subjected to a western blot with the indicated antibodies. LC3-I and LC3-II were normalized to β-actin and the ratio of LC3-II/LC3-I was obtained. The quantification of 3 independent experiments is shown in bar graphs (mean ± SEM). ***p < 0.001, and **p < 0.01, indicates that the samples are statistically different compared to control (BSA) samples. The original images are presented in Supplemental Figure S1A–C. The dotted lines in the Supplemental figures indicate the cropped area.

SFA causes stressful conditions, including inflammation and endoplasmic reticulum (ER) stress^25,26^. Since autophagy is one of the stress–response processes, we examined whether SFA regulates autophagy in human endothelial cells. Palmitic acid (PA), the most abundant SFA in the serum, increased the accumulation of microtubule-associated protein light chain 3 (LC3-II, a membrane component of autophagosome) in a time dependent manner (Fig. 1A). The LC3-II protein abundance was increased approximately 5-fold (p < 0.001) within 8 h treatment with 200 µM PA. The accumulation of LC3-II reached the maximum at the concentration of 100 μM PA (Fig. 1B). Because the lipidation of LC3 (e.g. LC3-II formation) indicates the steady-state level of autophagosome^33^, the result indicates that the amount of autophagosome was increased by palmitate. To examine whether the accumulation of LC3-II is a SFA specific response, the cells were treated with lauric acid (C12), another SFA, or oleic acid (C18), a monounsaturated fatty acid. Accumulation of LC3-II was observed when the cells were treated with lauric acid, but not with oleic acid (Fig. 1C). This result suggests that SFAs (e.g. lauric acid and palmitic acid) but not unsaturated fatty acid (e.g. oleic acid) cause the accumulation of autophagosome.

### Beta-oxidation regulates autophagic flux

**Figure 2:**
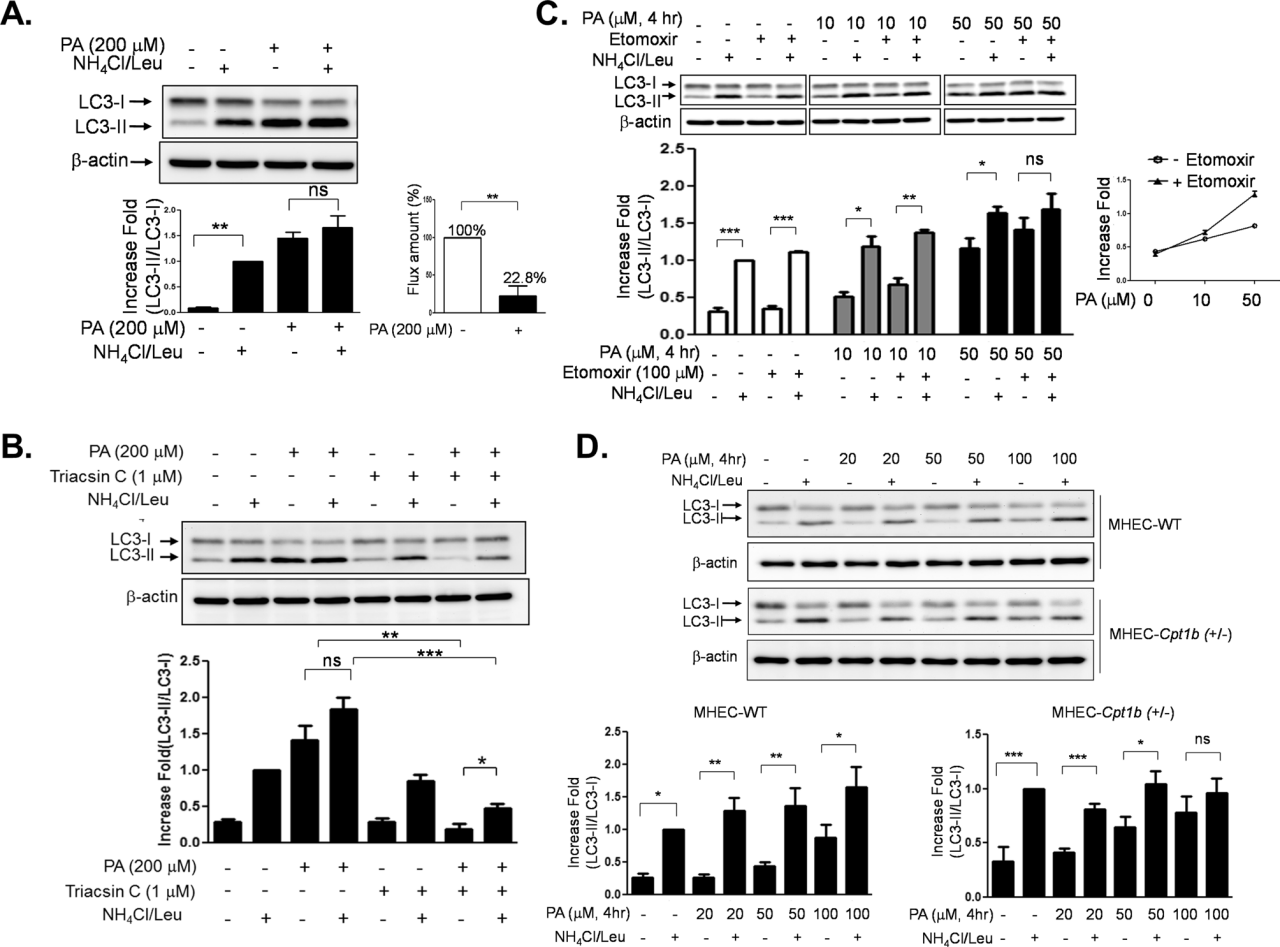
Beta-oxidation regulates autophagic flux. (**A**) Human endothelial cells were serum starved for 2 h, and then treated with PA (200 μM, 4 h). The cells were treated without or with NH_4_Cl (20 mM)/Leu (200 μM) 1 h prior to cell harvest, and the cell lysate was analyzed by a western blot for LC3-II formation. The differences in the ratio of LC3-II/LC3-I between the absence and the presence of NH_4_Cl/Leu were calculated for the indication of autophagic flux. The quantification of autophagic flux was calculated by setting the BSA-treated samples as 100%. (**B**) Human endothelial cells were pretreated with triacsin C (1 μM) for 30 min, and then treated with PA (200 μM, 4 h) followed by the treatment without or with NH_4_Cl/Leu 1 h prior to cell harvest. (**C**) Human endothelial cells were pretreated with etomoxir (100 μM) for 30 min before the treatment with PA (200 μM, 4 h). Next, the cells were treated without or with NH_4_Cl (20 mM)/Leu (200 μM) 1 h prior to cell harvest. Impairment of autophagic flux was more severe when β-oxidation was blocked by etomoxir. The differences in the ratio of LC3-II/LC3-I between the absence and the presence of NH_4_Cl/Leu were calculated for the indication of autophagic flux. The quantification of autophagic flux was calculated by setting the BSA-treated samples as 100%. (**D**) Mouse primary cardiac endothelial cells (MHEC) were isolated from WT or *Cpt1b* (+/−) mice, and then the cells were treated with PA at the indicated concentration for 4 h. The cells isolated from *Cpt1b* (+/−) demonstrated more severe impairment of PA-induced autophagic flux compared to WT mice. The quantification of 3 independent experiments is shown in bar graphs (mean ± SEM). ***p < 0.001, **p < 0.01, and *p < 0.05, indicate that the samples are statistically different between the indicated samples. The original images are presented in Supplemental Figure S2A–D. The dotted lines in the Supplemental figures indicate the cropped area.

Since lysosomal degradation is the final process of autophagy, we examined whether PA regulates autophagic flux (a net change between autophagosome formation and degradation). The autophagic flux was assessed by subtracting the ratio of LC3-II/LC3-I in the NH_4_Cl/leupeptin (Leu)-untreated cells (e.g. steady-state LC3-II) from the ratio of LC3-II/LC3-I in the NH_4_Cl/Leu-treated cells (the total amount formed during the given time). The amount of degraded LC3-II was much larger in the vehicle (BSA)-treated samples than in the PA-treated samples (22% of BSA-treated samples) (Fig. 2A), indicating that the accumulated LC3-II was due to the impaired lysosomal degradation of LC3-II. This impairment of autophagic flux was blunted by triacsin C, an inhibitor of acyl-CoA synthetases (ACSLs, enzymes that convert FA to fatty acyl-CoA), suggesting that long chain acyl-CoA or its metabolic products may block the lysosomal function^34^. The data suggest that the abundance of intracellular acyl-CoA regulates autophagic flux (Fig. 2B). Next, we examined whether β-oxidation regulates autophagic flux because most of the intracellular acyl-CoA is metabolized by mitochondrial β-oxidation. Mitochondrial fatty acid oxidation was blocked by using etomoxir, an inhibitor of carnitine pamitoyltransferase-1 (CPT1). In this experiment, submaximal concentrations of PA (10 μM and 50 μM) instead of saturating dose (200 μM) were used to observe the effect of etomoxir on autophagic flux. Blocking β-oxidation by etomoxir exacerbated the impairment of autophagic flux (Fig. 2C). To further corroborate this result, we examined the effect of reduced Cpt1b on autophagic flux in the primary mouse cardiac endothelial cells (MHEC) isolated from *Cpt1b *(+/−) heterozygous mice. Consistent with Fig. 2C, the autophagic flux was only partially impaired by SFA in the MHECs isolated from wild type (WT) mice, whereas the autophagic flux was completely blocked by SFA in the MHECs isolated from *Cpt1b* (+/−) mice. Altogether, these results indicate that a reduction in mitochondrial fatty acid oxidation contributes to the accumulation of LC3-II and autophagosome degradation (Fig. 2D).

### Metformin ameliorates the SFA-induced impairment of autophagic flux and reduces the accumulation of lipid droplet

**Figure 3:**
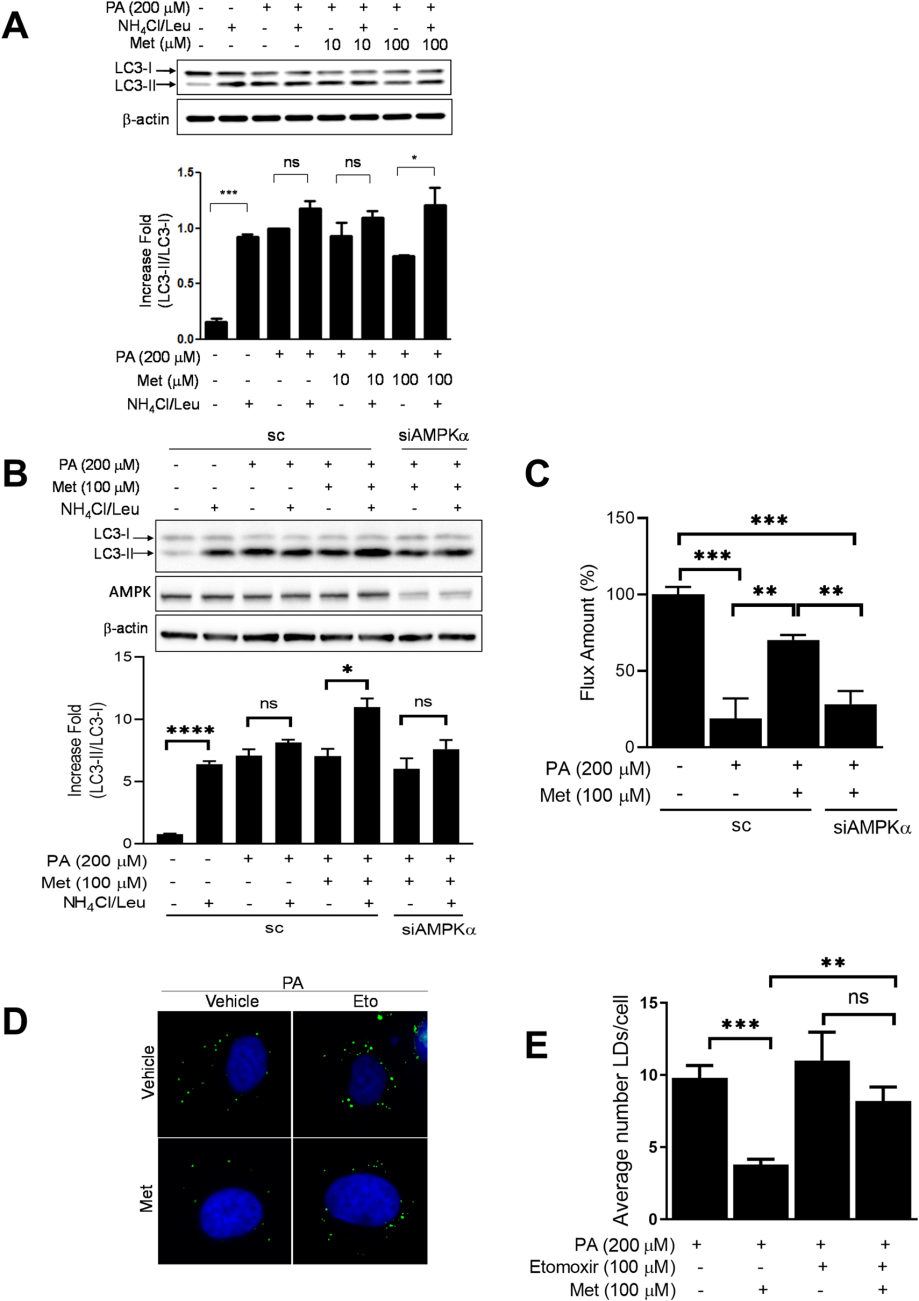
Metformin ameliorates the SFA-induced impairment of autophagic flux and reduces the accumulation of lipid droplets. (**A**) Human endothelial cells were serum starved for 2 h, and then pretreated with metformin (Met, 100 μM) for 30 min followed by treatment with PA (200 μM, 4 h). The cells were treated without or with NH_4_Cl (20 mM)/Leu (200 μM) 1 h prior to cell harvest. The cell lysates were subjected to a western blot with the indicated antibodies. Pre-treatment with metformin enhanced autophagic flux. (**B**) Human endothelial cells were transfected with siRNA for scrambled (sc) or AMPKα for 48 h. The cells were serum starved for 2 h and then pretreated with metformin (100 μM) for 30 min followed by treatment with palmitate (200 μM, 4 h). The cells were treated without or with NH_4_Cl (20 mM)/Leu (200 μM) 1 h prior to cell harvest, and then cell lysates were harvested. The cell lysates were subjected to a western blot with the indicated antibodies. (**C**) Autophagic flux was calculated by setting the BSA-treated cells as 100%. (**D**) Endothelial cells were pre-treated with etomoxir (100 μM) 30 min prior to the treatment with PA (200 μM, 4 h) and metformin (100 μM). (**E**) Inhibition of β-oxidation by etomoxir blunted the effects of metformin. LD numbers were obtained by using Image J as described in “Materials and methods”. The quantification of three independent experiments is shown in bar graphs (mean ± SEM). ***p < 0.001, **p < 0.01, and *p < 0.05 indicate that the samples are statistically different between the indicated samples. The original images are presented in Supplemental Figure S3A,B. The dotted lines in the Supplemental figures indicate the cropped area.

Metformin promotes lipid metabolism in skeletal muscle, liver, and brown adipose tissue^35–37^. However, the effect of metformin in SFA-induced impairment of autophagy in endothelial cells is unknown. We examined whether metformin can improve autophagic flux. Pre-treatment with metformin decreased the accumulation of LC3-II and ameliorated the SFA-induced impairment of autophagic flux (Fig. 3A). The results indicate the ability of metformin to improve autophagic flux. Next, we examined whether AMPKα is involved in the effect of metformin on autophagic flux. Human endothelial cells were transfected with siRNA for AMPKα or scrambled, and then the cells were treated with or without SFA in the presence or absence of metformin. The knock-down of AMPKα blunted the effect of metformin on autophagic flux by 42% (from 72.2 to 28.2%) (Fig. 3B,C). The results suggest that metformin improves autophagic flux through an AMPKα-mediated mechanism. Since the accumulation of intracellular lipid droplets (LD) is regulated by autophagy^3,21,38^, we investigated whether metformin regulates the accumulation of LD. Interestingly, pre-treatment with metformin decreased the abundance of LD (Fig. 3D,E). Moreover, the blocking β-oxidation by etomoxir significantly suppressed the effect of metformin on reducing LD accumulation (Fig. 3D,E). This suggests that the cytosolic amount of acyl-CoA may play an important role in not only autophagic flux but also LD accumulation.

### Metformin reduces LD by enhancing the degradation of LD, but not by inhibiting the formation of LD

**Figure 4:**
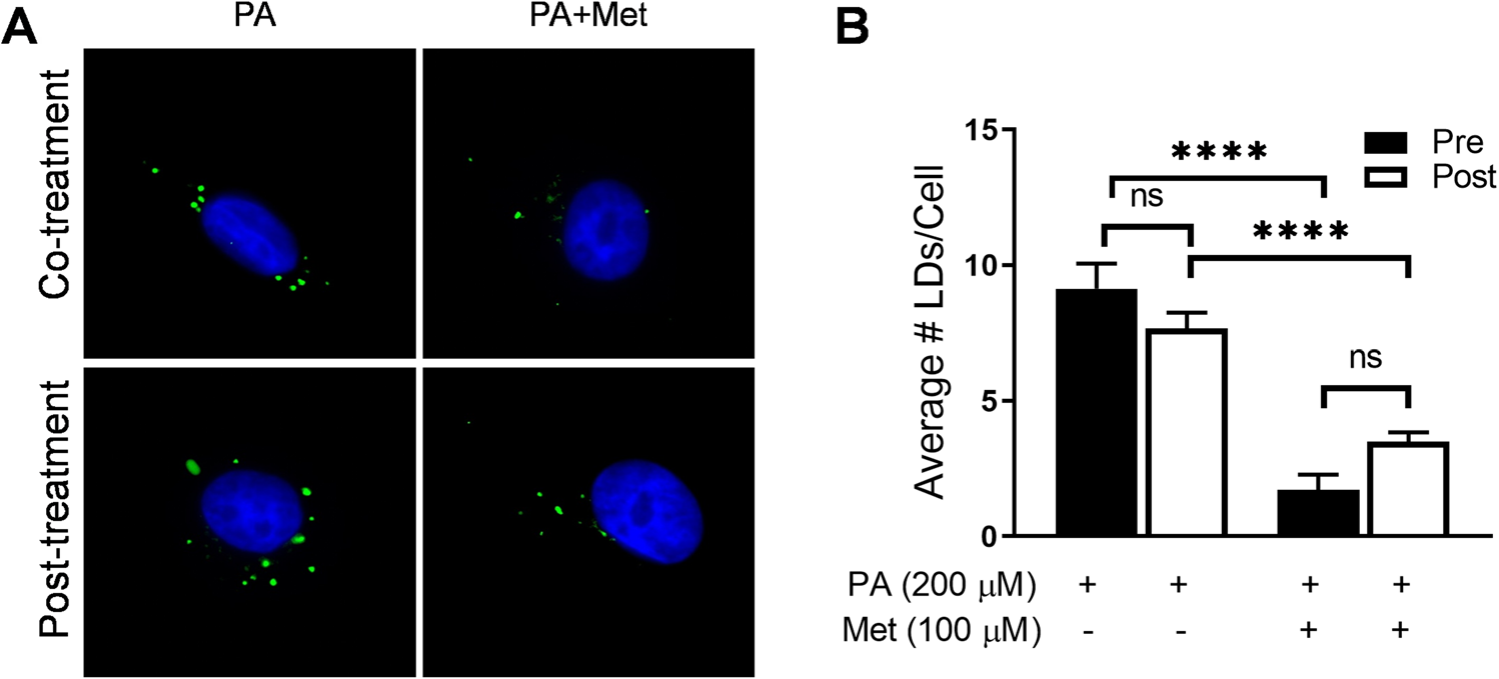
Metformin reduces LD by enhancing the degradation of LD, but not by inhibiting the LD formation. (**A**) For the co-treatment experiment, endothelial cells were incubated without or with metformin (100 μM) concurrently with PA (200 μM) for 4 h. For the post-treatment experiments, endothelial cells were incubated without or with PA (200 μM) for 4 h, and then the cells were washed with PBS to remove PA. Then the cells were treated without or with metformin (100 μM) for another 4 h. The cells were fixed with paraformaldehyde. Cells were stained with BODIPY 493/503 (green), and nuclei were stained with Hoechst 33342 (blue). The number of LD was counted and the average number of LD per cell was calculated. (**B**) Five to ten microscopic fields per condition were obtained and quantified by using Image J. The quantification of LD numbers is shown in bar graphs. Data are mean ± SEM (*p < 0.05).

We examined whether metformin increases the degradation or reduces the formation of LD. Endothelial cells were treated with metformin during or after the LD was formed. The number of LD was reduced to a similar extent of ≃ 86% under both conditions (Fig. 4A,B). The result suggests that metformin enhances the degradation of LD rather than inhibition of LD formation. Thus, the reduction of LD by metformin seems to be mainly mediated by autophagic degradation.

### Impairment of lysosomal dysfunction causes accumulation of lipid droplet

**Figure 5:**
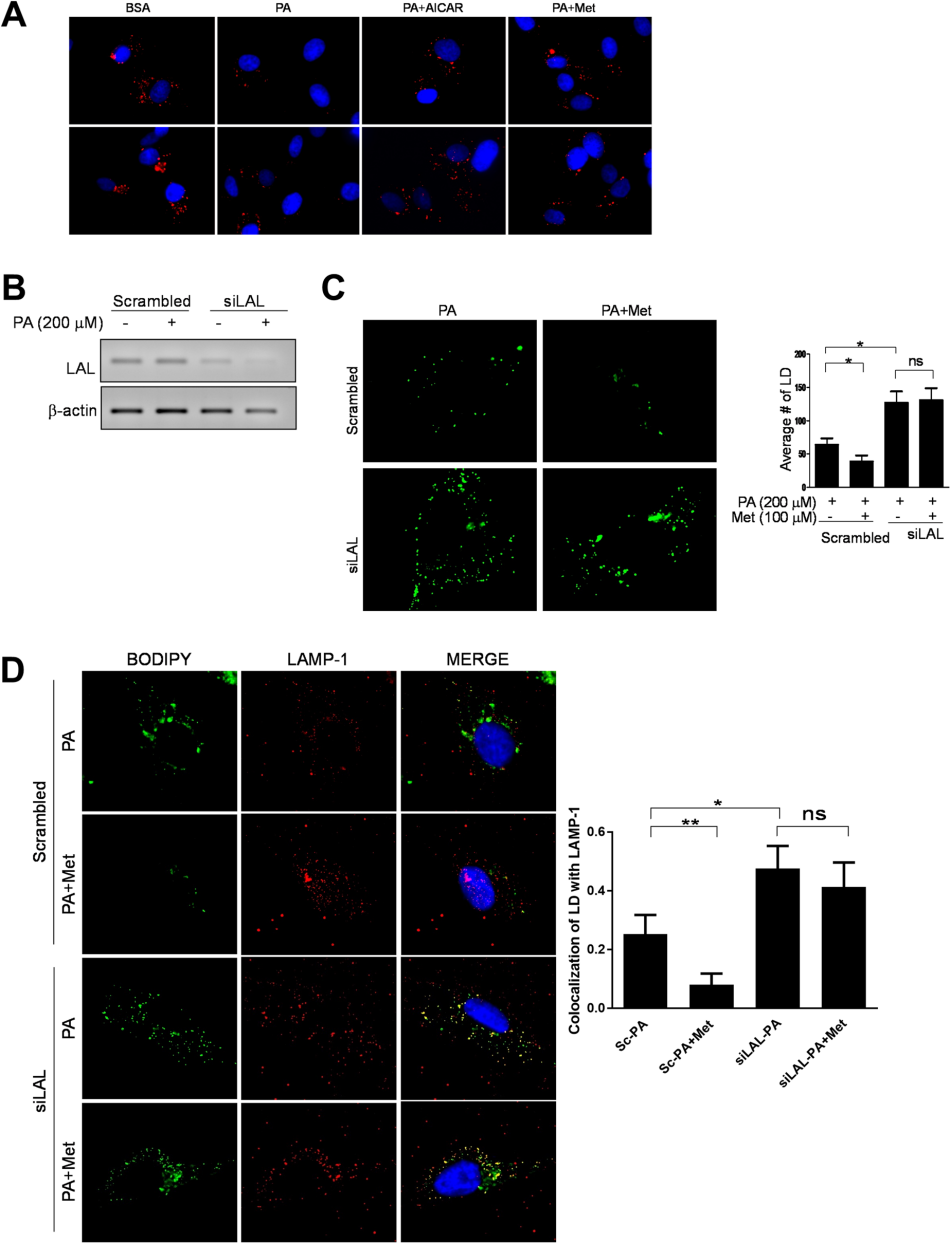
Impairment of lysosomal dysfunction causes the accumulation of LD. (**A**) Endothelial cells were pre-treated with either AICAR (200 μM) or metformin (100 μM) 30 min prior to PA (200 μM). The acidic lysosome was stained with a lysotracker (red), and the nucleus was stained with Hoechst 33342 (blue). Treatment with PA elevated pH of the lysosome and pre-treatment with AICAR or metformin restored the acidic PH of the lysosome. (**B**) Endothelial cells were transfected with scrambled or siRNA for LAL. siRNA for LAL reduced the expression of LAL. (**C**) The knock-down of LAL blunted the effect of metformin in LD reduction. Human endothelial cells were transfected with siRNA for scrambled or LAL. Endothelial cells were pre-treated without or with metformin (100 μM) 30 min prior to PA (200 μM). LD was stained with BODIPY (green). The number of LD was quantified by using Image J and is shown in bar graphs (mean ± SEM). ****p < 0.0001, ***p < 0.001, **p < 0.01, and *p < 0.05 indicate that the samples are statistically different between the indicated samples. (**D**) The knock-down of LAL increased the co-localization of lipid droplet (BODIPY, green), with LAMP-1 (lysosome marker, red). Six microscopic fields per condition were obtained and quantified by using Image J. The quantification of colocalization of LD with LAMP-1 is quantified with Image J and is shown in bar graphs (mean ± SEM). **p < 0.01 and *p < 0.05, indicates that the samples are statistically different between the indicated samples. The full-length agarose gel is presented in Supplemental Figure S5A, and the inverted image is presented in Supplemental Figure S5B. The dotted lines in the Supplemental figures indicate the cropped area.

Increased pH in the lysosome causes lysosomal dysfunction and is associated with impaired autophagic flux^39^. Indication of lysosomal pH was assessed by a lysotracker. Treatment with PA elevated the lysosomal pH, which was restored by AICAR (an AMPK activator) or metformin (Fig. 5A). This suggests that metformin may ameliorate the SFA-induced lysosomal dysfunction by regulating lysosomal pH. Lysosomal acid lipase (LAL) is a lysosomal specific lipase, which contributes to lipophagy^40,41^. To examine whether LAL is involved in the reduction of LD by metformin, LAL was knocked down by using siRNA for LAL (Fig. 5A). We confirmed that LAL was reduced by more than 80% by siRNA transfection (Fig. 5B). The ability of metformin to reduce the number of LD was blunted by LAL knock-down (Fig. 5C). The co-localization of LD with LAMP-1, a lysosomal marker protein indicates that LD is degraded by autophagolysosome^21^. Interestingly, treatment with metformin decreased the co-localization of LD with LAMP-1 by 68.3%, which suggests that LD was degraded by metformin in cells transfected with scrambled siRNA. The knock-down of LAL increased the co-localization of LD with LAMP-1 by twofold, both in the presence and absence of metformin compared to the fatty acid-treated control cells (Fig. 5D). The data suggests that LD degradation by metformin is dependent on LAL activity.

### Metformin suppressed leukocyte adhesion by enhancing lysosomal function

**Figure 6:**
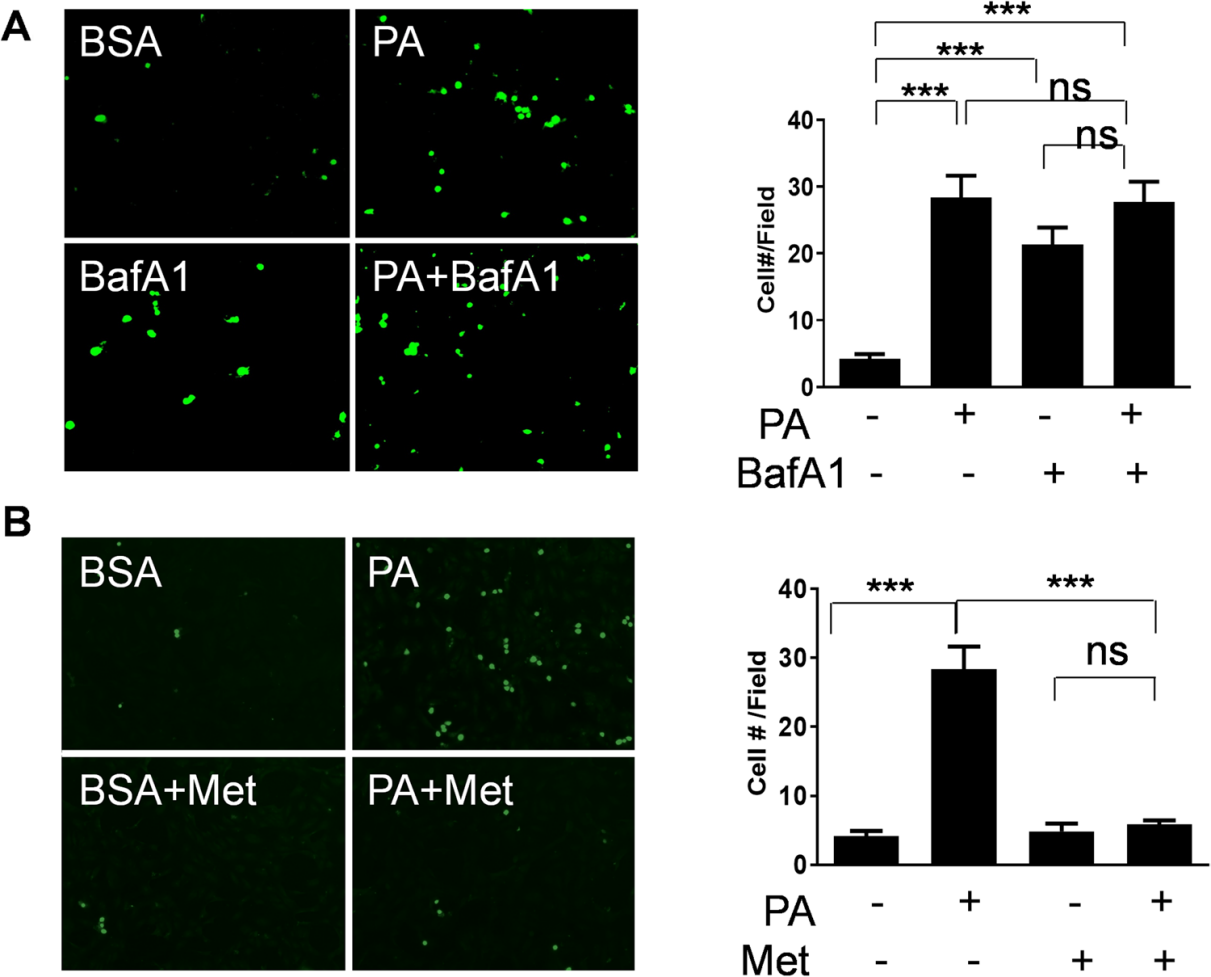
Metformin suppressed leukocyte adhesion by enhancing lysosomal function. (**A**) Human endothelial cells were grown to confluency and treated with PA (200 μM) with or without bafilomycin A1 (BafA1, 10 μM) for 4 h. Calcein-AM labeled human monocytes (U937) were co-cultured with endothelial cells for 30 min. The non-adherent cells were then washed, and the bound cells were counted (5 fields each condition). (**B**) Human endothelial cells were grown to confluency and then treated with PA (200 μM) with or without metformin (Met, 100 μM) for 4 h. Calcein-AM labeled human monocytes (U937) were co-cultured with endothelial cells for 30 min. The non-adherent cells were then washed, and the bound cells were counted (5 fields each condition). The quantification of adherent cell numbers is shown in bar graphs (mean ± SEM). ***p < 0.001 indicates that the samples are statistically different between the indicated samples.

SFA stimulates a pro-inflammatory response in vascular endothelial cells^25,26,42^. Thus, we examined whether autophagy plays a role in the SFA-induced inflammatory response. The co-culture of human monocyte with endothelial cells was performed and the adhesion of human monocyte was evaluated with or without inhibition of autophagy. Treatment with bafilomycin A1 increased monocyte adhesion by fivefold, which is comparable to the results from the treatment with PA (Fig. 6A). Furthermore, the treatment with metformin decreased PA-induced monocyte adhesion by 79.5% (Fig. 6B). The result suggests that metformin-stimulated autophagic flux may contribute to the anti-inflammatory action of metformin.

## Discussion

**Figure 7:**
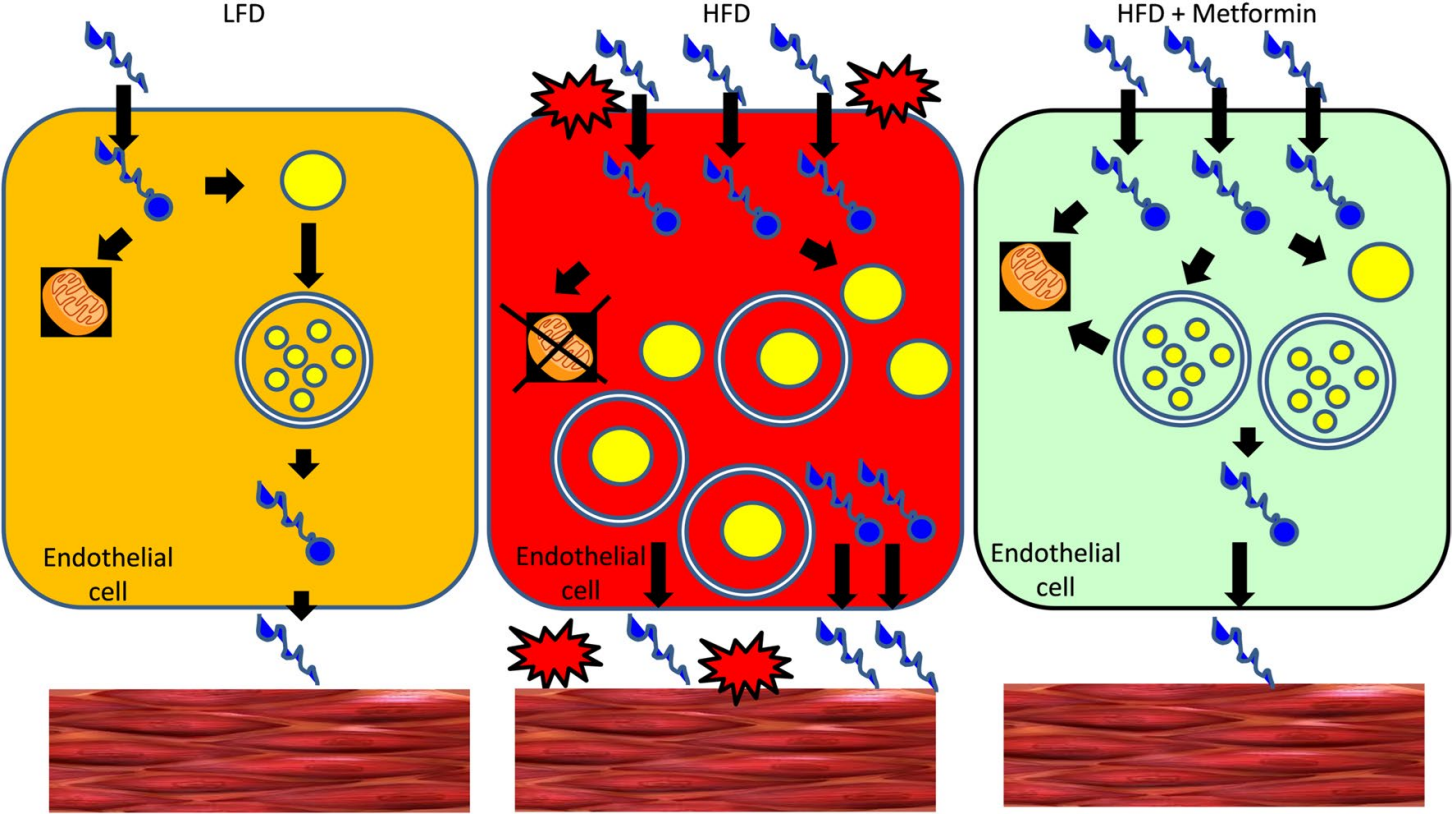
A proposed mechanism by which metformin reduces the accumulation of lipids in endothelial cells. Low levels of fatty acids [low fat diet (LFD)] are metabolized by mitochondria or transported to peripheral tissues (left). When an excess amount of fatty acids are provided [high fat diet (HFD)], mitochondrial dysfunction, and impaired autophagy (lipophagy) reduces fatty acid oxidation and increases the intracellular pool of fatty acids/acyl-CoA, which leads to accumulation of lipid droplets. This stressful condition stimulates pro-inflammatory responses (middle). Treatment with metformin stimulates fatty acid oxidation and autophagy (lipophagy) which reduces the accumulation of lipid droplets and the intracellular pool of fatty acids. This action of metformin contributes to the reduction of pro-inflammatory response (right).

Autophagy is an essential catabolic process for normal cellular physiology and is activated by stressful conditions including starvation and over-nutrition as a survival mechanism^7^. Human subjects with metabolic dysfunction, such as diabetes and obesity, have higher fatty acid levels with increased inflammatory responses^43,44^. These inflammatory responses are associated with cardiovascular diseases, neurodegeneration, and metabolic syndrome^44–47^. Although the involvement of autophagy in normal immune function and inflammation is manifold, a growing body of evidence indicates that autophagy is one of the crucial mechanisms that regulate inflammatory responses^34^. We previously demonstrated that SFA causes pro-inflammatory responses through a toll-like receptor-mediated mechanism in vascular endothelial cells^25,26^. However, the link between SFA-induced inflammatory response and autophagy in endothelial cells is largely unknown. Metformin is the first line of treatment for T2DM and is known to have additional beneficial effects in patients with cardiovascular disease or cancer^29,48,49^. In the present study, we demonstrate the anti-inflammatory effect of metformin which is mediated by the increased autophagic flux in endothelial cells. This presents a novel mechanism by which metformin protects cardiovascular function in patients with diabetes and dyslipidemia (Fig. 7).

Subjects with obesity and/or diabetes have elevated circulating lipid levels that cause endothelial dysfunction^25,26,50–52^. The mechanisms by which SFA causes endothelial dysfunction are increased reactive oxygen species inflammatory response, and endoplasmic reticulum stress^25,51–54^. We demonstrate that SFA but not monounsaturated fatty acid increases the accumulation of LC3-II (Fig. 1C). Although lauric acid is shorter than PA, lauric acid induces pro-inflammatory response as potent as palmitic acid in macrophage cell line^55,56^. Likewise, lauric acid may cause more stress than oleic acid. These cellular stress conditions may affect autophagy, which could be a novel mechanism for endothelial dysfunction and vascular inflammation^57–59^. Therefore, it is plausible that SFA may affect the autophagy process. In fact, SFA increases autophagosome formation, but the autophagic degradation (flux) is impaired due to the elevated lysosomal pH (Fig. 5). This impairment of autophagic flux may contribute to ectopic lipid accumulation, the fat deposit in non-adipose tissue. Since ectopic lipid accumulation is associated with metabolic syndrome, our findings suggest that SFA causes cardiovascular and metabolic dysfunction through impaired autophagic flux.

Most cytosolic fatty acids are ligated to coenzyme A (CoA) by acyl-CoA synthetases before the fatty acyl-CoAs undergo β-oxidation or further cellular utilization^60^. Inhibition of fatty acyl-CoA synthesis by triacsin C reversed the PA-induced impairment of autophagic flux (Fig. 2B). Because there are several isotypes expressed in endothelial cells, it would be interesting to identify a specific ACSL isotype that plays an important role in autophagic degradation. However, it is also possible that each isotype may compensate for the functions of each other. To clarify this issue, further studies are necessary. Inhibition of β-oxidation reduces the flux amount (Fig. 2C). This result suggests that the cytosolic fatty acyl-CoA may contribute to the impairment of autophagic flux due to the abundance of mitochondria (Fig. 7). Moreover, the effect of β-oxidation on autophagic flux may be dependent on cell types, because the amount of cytosolic acyl-CoA is dependent on the number of mitochondria and its function. Because endothelial cells are not a mitochondria-rich cell type compared to skeletal muscle or cardiac tissue, endothelial cells may be more sensitive to FA-induced impairment of autophagy.

Since fatty acyl-CoA is a component of triglycerides (TG), increased the cytosolic fatty acyl-CoA is associated with TG synthesis, which makes lipid droplets. When the cells are undergoing the anabolic cycle, the lysosomal degradation is likely to be inhibited. Deficiency of long chain fatty acyl-CoA synthetase 1 increases dysfunctional mitochondria in cardiac tissue^61^. Due to mitochondrial dysfunction, fatty acid utilization is impaired while glucose utilization is enhanced^61^. Therefore, the increased glucose utilization may stimulate the expression of ITM2A, which is a negative regulator of vacuolar ATPase (V-ATPase)^62^. Thus, V-ATPase may be inhibited by accumulated fatty acyl-CoA and the increased ITM2A, which causes the elevation of lysosomal pH. The detailed mechanisms by which SFA regulates V-ATPase are remained to be further investigated.

We observed that the treatment with etomoxir increased the accumulation of LD in the presence of metformin (Fig. 3D, E). This may be due to the effect of metformin on the LD accumulation is by increasing β-oxidation or by some other unknown degradation pathway. Furthermore, the knock-down of LAL abolished the effect of metformin on LD accumulation (Fig. 5C). This suggests that metformin reduces LD accumulation at least in part through autophagic degradation.

The pharmacological concentration of metformin reaches 40–70 μM in the portal vein, and 10–40 μM in circulation^63^. The primary action of metformin is inhibition of hepatic gluconeogenesis by both AMPK-dependent and -independent mechanisms^29,64^. Metformin suppressed gluconeogenesis through activation of AMPK at lower concentrations (< 250 μM), while decreasing ATP levels at higher concentrations (> 250 μM). High concentrations of metformin (2–5 mM) inhibit respiratory chain complex 1 in hepatocytes, which leads to an increased in the AMP/ATP ratio by an AMPK-independent mechanism^64,65^. We used a low concentration of metformin (100 μM) in the present study. Thus, the effect of metformin in our study seems to be through an AMPK-dependent mechanism rather than by increasing AMP/ATP ratio via inhibition of respiratory chain complex 1 (Fig. 3B,C). Also, a previous study demonstrates that metformin directly activates V-ATPase^66^, which is consistent with our result (Fig. 5). This suggests that the protective effect of metformin in fatty acid-induced lipotoxicity may be mediated by the stimulation of AMPK-dependent autophagic flux.

In the present study, we demonstrate the effects of metformin on autophagy and its role in LD accumulation in vascular endothelial cells (Figs. 3, 4, 5). These suggest a novel mechanism by which metformin protects cardiovascular functions from lipotoxicity and dyslipidemia (Fig. 7). In addition to anti-diabetic action, metformin has beneficial effects on cardiovascular functions, including improvement of endothelial function and prevention of heart failure and atherosclerosis^28,31,67–71^. Activation of AMPK is one of the major mechanisms for the vasculoprotective effects of metformin^72^. Further study is warranted on the involvement of AMPK and eNOS in metformin-stimulated autophagic flux and reduced LD accumulation.

Inhibition of autophagy increased monocyte adhesion which was suppressed by metformin (Fig. 6). We and others reported that SFA causes endothelial dysfunction and pro-inflammatory responses through toll-like receptor (TLR)-mediated mechanisms^25,26,42^. Since metformin has an anti-inflammatory effect and attenuates TLR signaling in liver, skeletal muscle, and heart, it would be of interest to investigate whether metformin-induced autophagic flux affects innate immune response^73–76^. The deficiency of autophagy in endothelial cells exacerbates atherosclerosis^77^. This suggests the impaired autophagic flux by SFA may contribute to the development of atherosclerosis.

In the current study, we demonstrate that SFA causes accumulation of LD and pro-inflammatory responses and that metformin ameliorates the adverse effects of SFA through an autophagy-dependent mechanism. In conclusion, autophagic flux is a novel mechanism by which metformin protects vascular endothelium from lipotoxic injuries.

## Materials and methods

### Materials

Anti-LC3 (#4599) and anti-β-actin (#3700) antibodies were obtained from Cell Signaling Technology (Danvers, MA, USA). NH_4_Cl, etomoxir, and leupeptin were purchased from Sigma-Aldrich (St. Louis, MO, USA). DsiRNA for AMPKα, LAL and scrambled dsiRNA were purchased from Integrated DNA Technologies (Coralville, IA, USA). Metformin, and bafilomycin A1 were obtained from Millipore Sigma (Burlington, MA, USA). Most of the compounds used in this study were dissolved in dimethyl sulfoxide, and we confirmed that the vehicle alone did not affect our results.

### Experimental animals

All mouse procedures and handlings were in accordance with the protocols approved by the Animal Use and Care Committee at The University of Alabama at Birmingham. *Cpt1b* (+/−) mice were a kind gift from Dr. Phllip A.Wood^78^. Heterozygous *Cpt1b* (+/−) mice and WT littermates in C57BL/6J background were used. Homozygous *Cpt1b* knockout mouse is lethal, while heterozygous *Cpt1b* (+/−) mice do not show an overt abnormal phenotype. All animals were maintained in a temperature-controlled facility with a 12 h light–dark cycle. At 6 wk of age. Mice had ad libitum access to water and standard rodent diet, 7017 NIH-31 Mouse/Rat Sterilizable Diet (Harlan Laboratories, Indianapolis, IN, USA).

### Isolation of mouse primary heart endothelial cells

The mouse heart endothelial cells (MHEC) were isolated using a modification of previously described methods^79^. Briefly, after 6 wk old mice were euthanized by isoflurane inhalation, the heart was dissected, and then minced heart tissue was incubated in 2 mg/ml collagenase I/PBS (Worthington Biochemical Co., Lakewood, NJ, USA) for 45 min at 37 °C. The collagen treated hearts were triturated with the syringe attached a cannula, filtered through a cell strainer and washed twice with DMEM. The cells were isolated by using microbeads (Dynal beads M-450, Invitrogen, Carlsbad, CA, USA) coated with anti-CD31 (BD Biosciences, San Jose, CA, USA) in PBS with 0.1% BSA for 10 min. The isolated endothelial cells were seeded onto gelatin-coated plates in DMEM supplemented with 20% FBS, 45 μg/ml endothelial cell growth supplement (ECGS), 100 U/ml penicillin, 100 μg/ml streptomycin, and 10 U/ml of heparin. MHEC were used between passages 3 and 6.

### Endothelial cell culture and transfection

Maintenance and transfection of endothelial were performed as previously described^25,80^. Immortalized human endothelial cell line EA.hy926 cells (CRL-2922) were obtained from ATCC (Manassas, VA, USA), and maintained in DMEM low glucose media containing 10% FBS, penicillin (100 U/ml) and streptomycin (100 μg/ml). Cells were transiently transfected with 100 nM of siRNA duplex oligonucleotides (siRNA for LAL or scrambled) using Lipofectamine 2000 (Invitrogen) according to the manufacturer's instructions. Two days after transfection, cells were serum-starved for 2 h and then treated with BSA or PA as indicated in legends to figures.

### Preparation of palmitic acid

Preparation of PA was carried out as described by Mott et al*.*^81^. Briefly, 10.5% BSA (Fitzgerald, MA, USA) was dissolved in 25 mM HEPES/DMEM, and filtered (0.22 μM, Millipore, Burlington, MA, USA). Sodium palmitate was heated to be dissolved in water and rapidly added to warmed BSA solution. Then this BSA-conjugated PA, oleic acid, or lauric acid (Nu-Chek, Elysian, MA, USA) were added to the concentrations as indicated in the figure legends. We used endotoxin-free reagents and we tested all the reagents we used including BSA, PA, media and reagent diluents. We checked the endotoxin level of all the reagents we used in this study by Chromogenic Endotoxin Quantitation assay kit (Thermo Fisher, Waltham, MA, USA). Lower than 25 pg/ml of endotoxin is undetectable.

### Preparation of cell lysate and immunoblotting

Cells were briefly washed with ice-cold PBS after the indicated treatments. Preparation of cell lysate and immunoblotting were performed as described previously^21,26^. Cells were then scraped in lysis buffer containing 50 mM Tris (pH 7.2), 125 mM NaCl, 1% Triton X-100, 0.5% NP-40, 1 mM EDTA, 4 mM Na_3_VO_4_, 20 mM NaF, 1 mM Na pyrophosphate, and complete protease inhibitor cocktail (Thermo Fisher) as described previously^25^. Cell debris was pelleted by centrifugation at 17,000×*g* for 10 min at 4 °C. Supernatants were then boiled with Laemli sample buffer for 5 min and proteins were resolved by 12% SDS-PAGE, transferred to nitrocellulose membranes, and immunoblotted with primary antibodies and peroxidase-conjugated secondary antibodies were incubated. The bands were visualized by using super signal chemiluminescent substrate (Thermo Fisher). Immunoblots were quantified by Image analyzer (Vision Works LS, UVP, LLC, CA, USA) and UVP Bioimaging Systems^25^.

### Immunocytochemistry

Immunocytochemistry was performed as described previously^21^. To visualize lipid droplets, cells grown on coverslips were fixed with 4% formaldehyde in PBS, and then BODIPY 493/503 (Thermo Fisher Scientific, Waltham, MA, USA) was stained for 30 min at room temperature. For immunofluorescent staining of cells, cells were treated as described in the figure legends. After stimulation, cells were fixed in 4% paraformaldehyde/PBS for 10 min and washed the cells with PBS. Cells were then permeabilized with 0.1% Triton X-100/PBS for 5 min and washed with PBS. Cells were blocked with 5% BSA/PBS for 1 h and then incubated with anti-LAMP-1 antibody in 5% BSA/PBS at 4 °C overnight. Cells were washed with PBS 3 times (5 min each) and then incubated with Alexa 555 conjugated-goat anti-rabbit IgG (Thermo Fisher, Waltham, MA, USA) for 1 h at room temperature. Images were acquired with an Axiovert fluorescence microscope (Carl Zeiss Ltd., Thornwood, NY, USA). Ten to fifteen cells were randomly selected from each treatment to calculate the average number of lipid droplets and the percentage of co-localization per cell. Quantification was performed using Image J software (NIH, MD, USA) and the percentage of co-localization was calculated by JACoP plugin of Image J. The data shown are from one representative experiment of three independent repeats.

### RT-PCR

The cells were treated as described in the figure legends. RT-PCR was performed as we described previously^25^. Total RNA was prepared by using TRIZOL (Invitrogen) according to the manufacturer’s instructions. cDNA was synthesized with 1 μg of total RNA by using Omniscript RT Kit (Qiagen, Valencia, CA, USA), and then the cDNA was subjected to semi-quantitative PCR analysis by using Hot Star Taq Master Mix kit (Qiagen). The PCR product was subjected to an agarose gel electrophoresis with fluorescent dye (Envirosafe DNA/RNA Stain, Helixx Technologies, Inc, Ontario, Canada) and the images were analyzed and quantified by an Image analyzer (Vision Works LS) and UVP gel imager. The primers for LAL 5′-CTGAAGGAGCTCTGTGGAAATC-3′ (forward), 5′-CCAGCAGGAGAATGTGTTGTAT-3′ (reverse); β-actin 5′CTGGCACCCAGCACAATGAAG-3′ (forward), 5′TAGAAGCATTTGGGGTGGACG-3′ (reverse) were used.

### Statistical analysis

Values are presented as mean ± standard error of the mean (SEM). Western blots were analyzed by one-way ANOVA.

### Ethical approval for animal study

Ethical approval is taken for using ‘mice’ in the study by the Animal Use and Care Committee at The University of Alabama at Birmingham.

## Supplementary information

Supplementary information

## Abbreviations

SFA: Saturated fatty acid
T2DM: Type 2 diabetes mellitus
AMPK: AMP-activated protein kinase
LC3: Microtubule-associated protein 1A/1B-light chain 3
ER: Endoplasmic reticulum
eNOS: Endothelial nitric oxide synthase
ULK1: Unc51-like kinase 1
CPT1: Carnitine palmitoyltransferase 1
LD: Lipid droplet
AICAR: 5-Aminoimidazole-4-carboxamide 1-β-d-ribofuranoside, Acadesine, N^1^-(β-d-ribofuranosyl)-5-aminoimidazole-4-carboxamide
LAL: Lysosomal acid lipase
LAMP-1: Lysosomal associated membrane protein 1
CoA: Coenzyme A
V-ATPase: Vacuolar ATPase
PA: Palmitic acid
