# Supplementary material for: Metformin reduces saturated fatty acid-induced lipid accumulation and inflammatory response by restoration of autophagic flux in endothelial cells
Source: Sci Rep. 2020 Aug 11;10:13523. doi: 10.1038/s41598-020-70347-w (PMC7419289; doi:10.1038/s41598-020-70347-w)
Supplement: Supplementary file 1 — Supplementary information [file 41598_2020_70347_MOESM1_ESM.pdf]

Supplemental Figures

Metformin reduces saturated fatty acid-induced lipid accumulation and inflammatory response by restoration of autophagic flux in endothelial cells

Hae-Suk Kim[1], Guang Ren[1], Teayoun Kim[1], Sushant Bhatnagar[1], Qinglin Yang[2], Young Yil Bahk[3], and Jeong-a Kim[1,*]

Comprehensive Diabetes Center and Department of Medicine, Division of Endocrinology, Diabetes, and Metabolism, University of Alabama at Birmingham, Birmingham, AL USA[1].; Department of Nutrition, University of Alabama at Birmingham, Birmingham, AL USA[2]; Department of Biotechnology, College of Biomedical and Health Science, Konkuk University, Chungju 27478, Republic of Korea [3]

*** Address correspondence to:**

Department of Medicine, Division of Endocrinology, Diabetes, and Metabolism, University of Alabama at Birmingham, Shelby 1214, 1825 University Blvd, Birmingham, AL 35294 USA

Tel; 205-934-4128;

Fax; 205-975-9372

E-mail:jakim@uab.edu

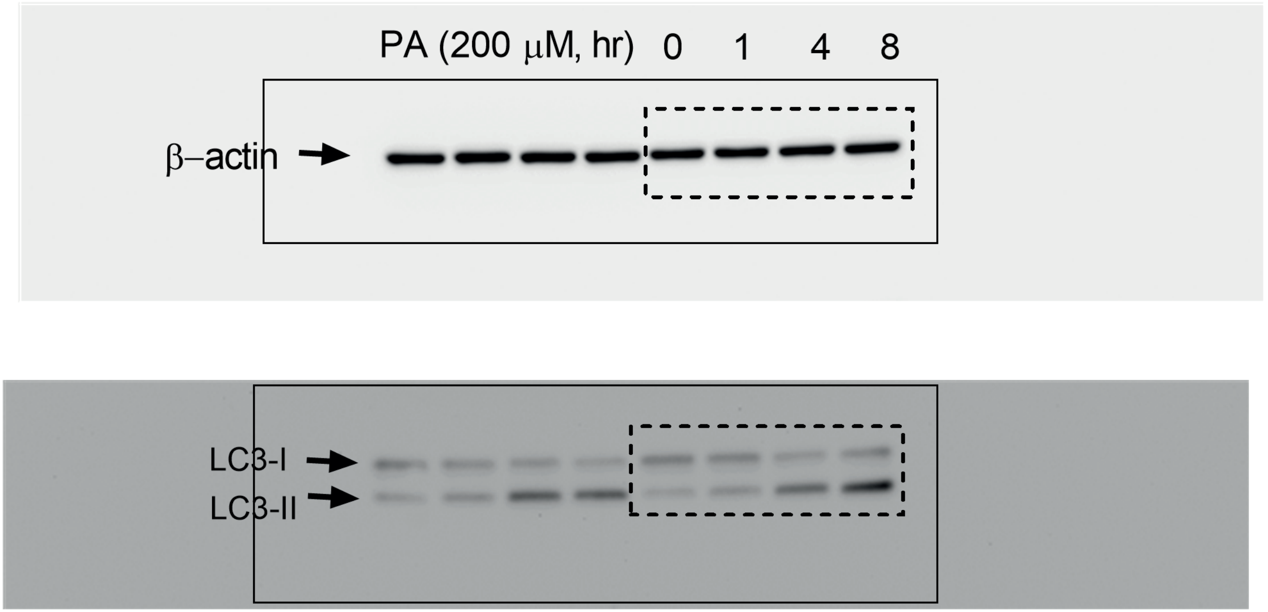

Supplemental Figure S1A. Orginal scans associated with Figure 1A
Dottted lines are the cropped area, and the solid lines are the edge of the blots.

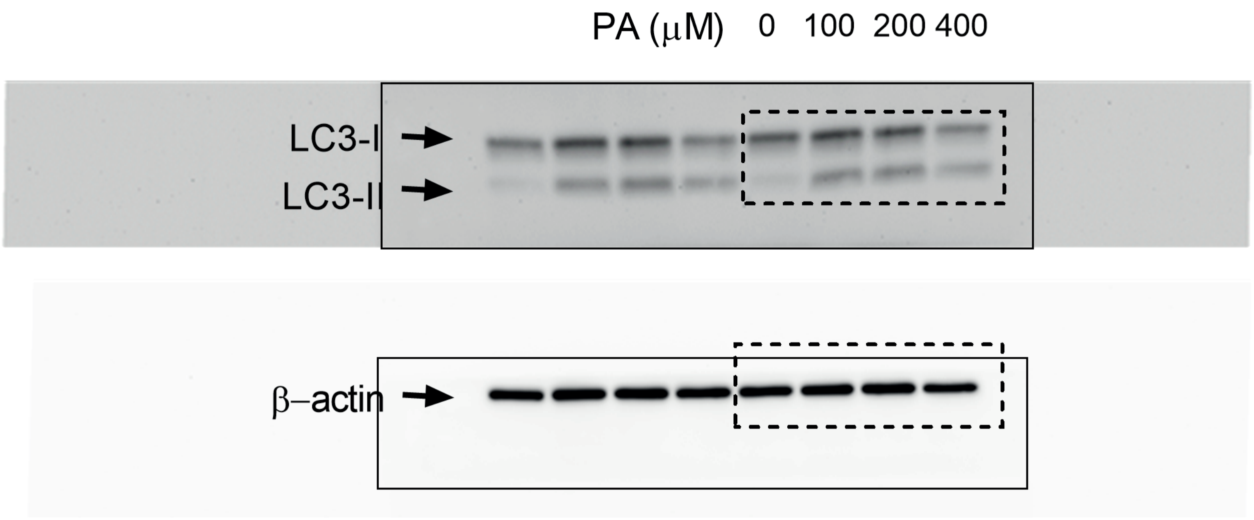

Supplemental Figure S1B. Orginal scans associated with Figure 1B. Dotttted lines are the cropped area, and the solid lines are the edge of the blots.

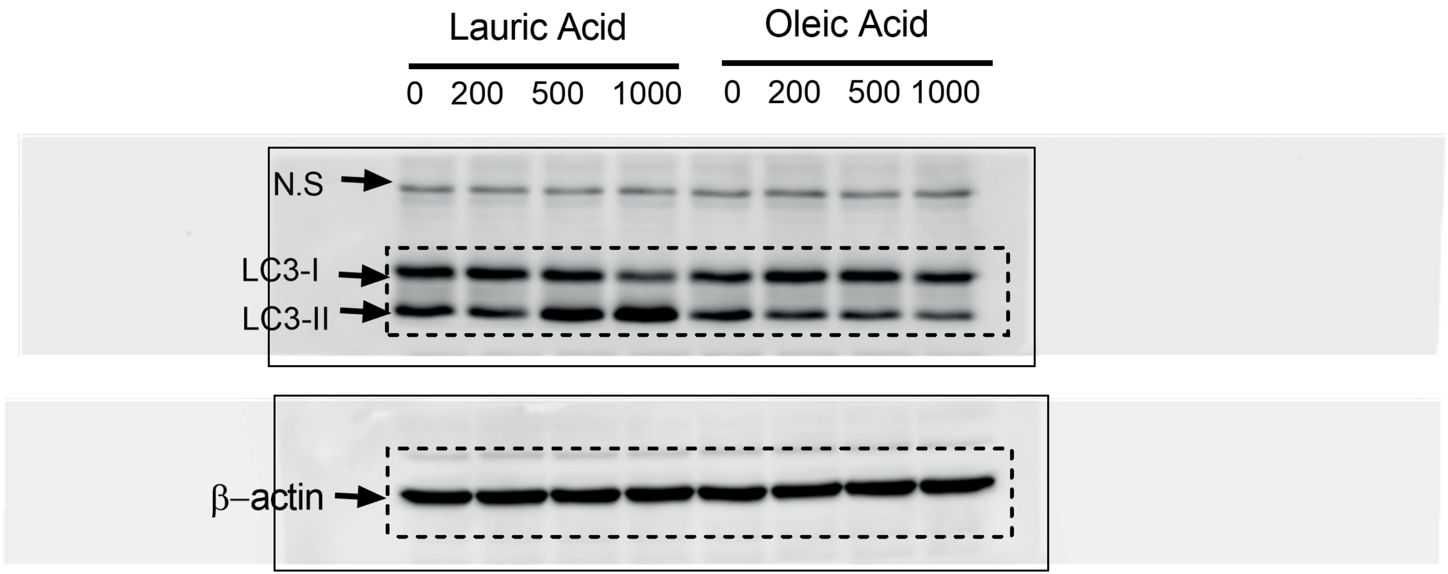

Supplemental Figure S1C. Orginal scans associated with Figure 1C. Dottted lines are the cropped area, and the solid lines are the edge of the blots.

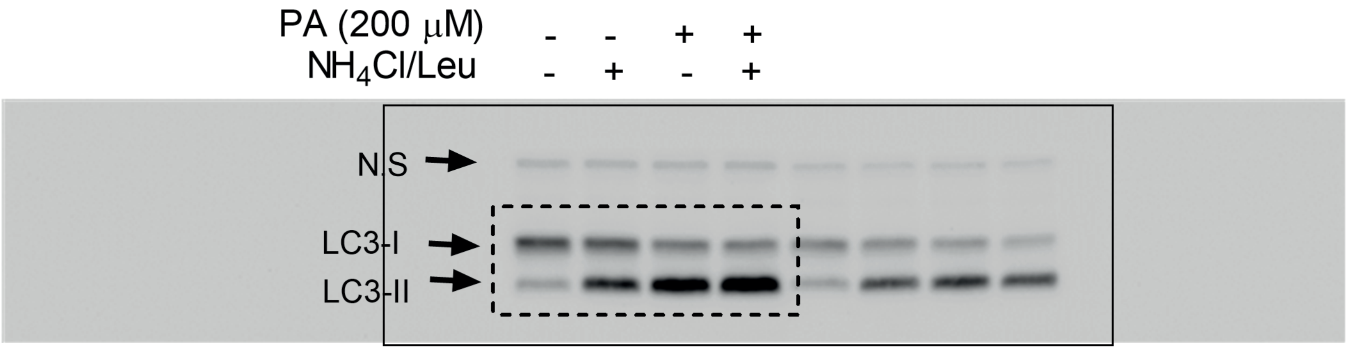

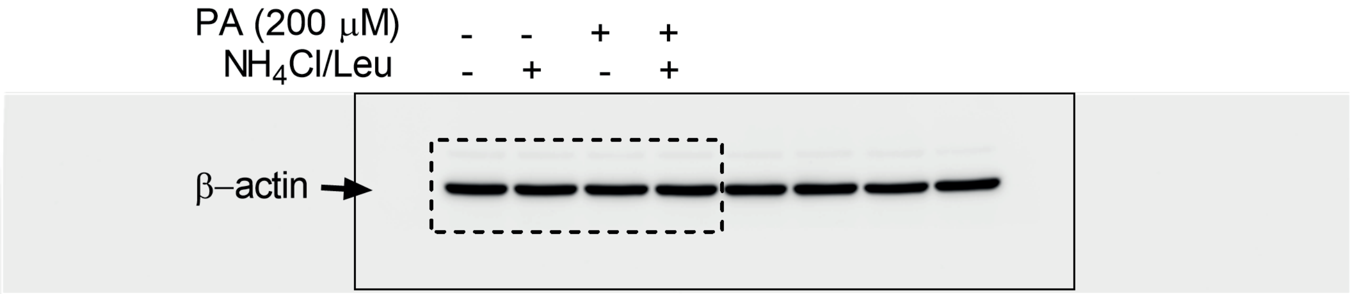

Supplemental Figure S2A. Orginal scans associated with Figure 2A. Dottted lines are the cropped area, and the solid lines are the edge of the blots.

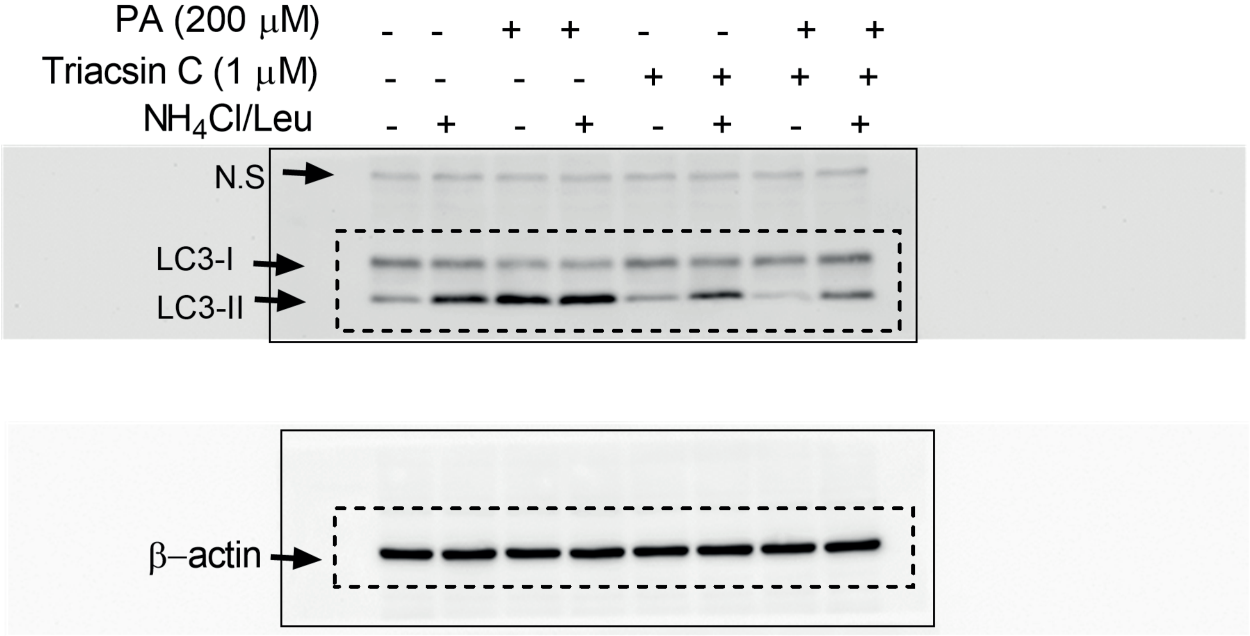

Supplemental Figure S2B. Orginal scans associated with Figure 2B. Dottted lines are the cropped area, and the solid lines are the edge of the blots.

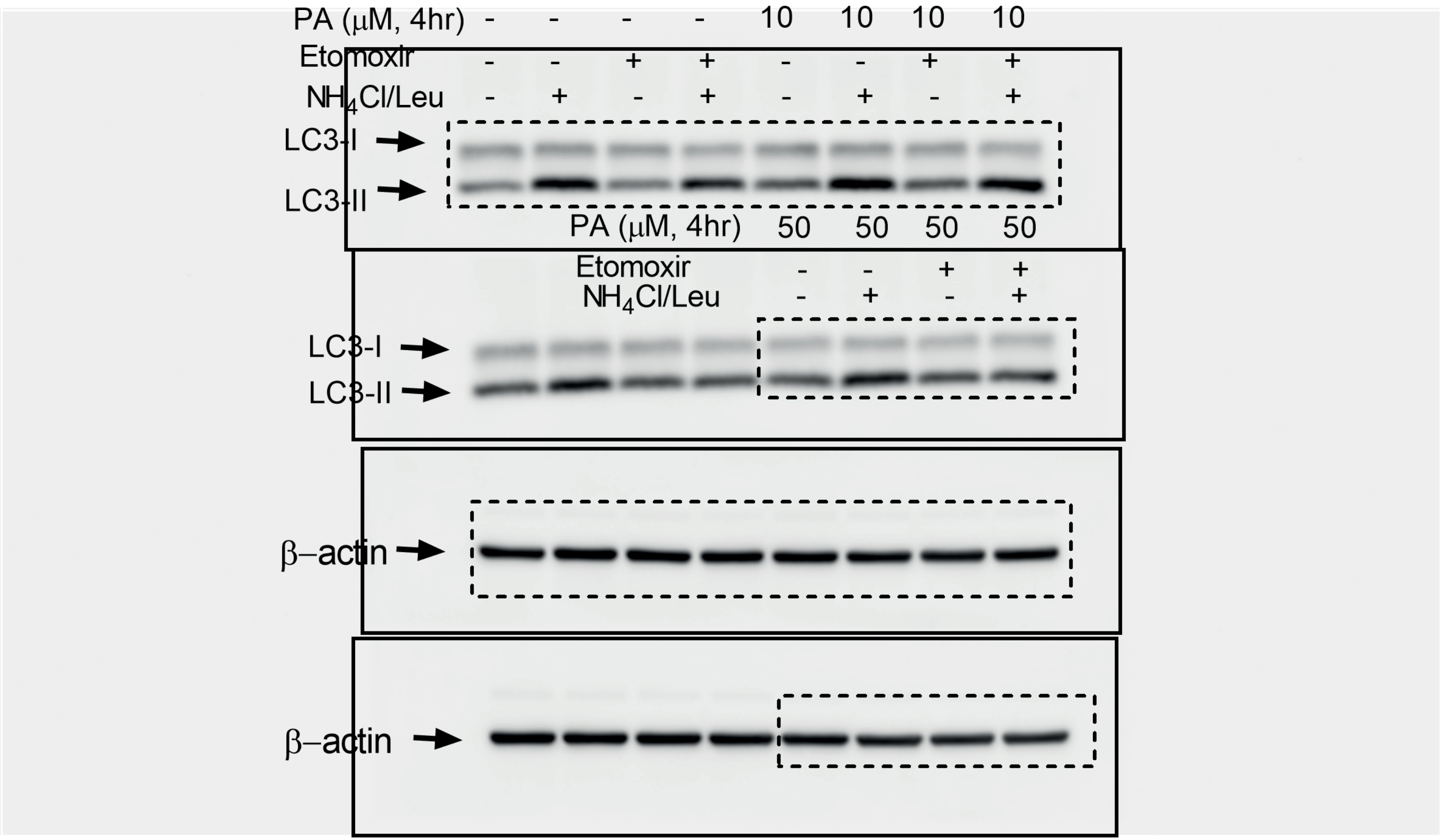

Supplemental Figure S2C. Orginal scans associated with Figure 2C.
Dottted lines are the cropped area, and the solid lines are the edge of the blots.

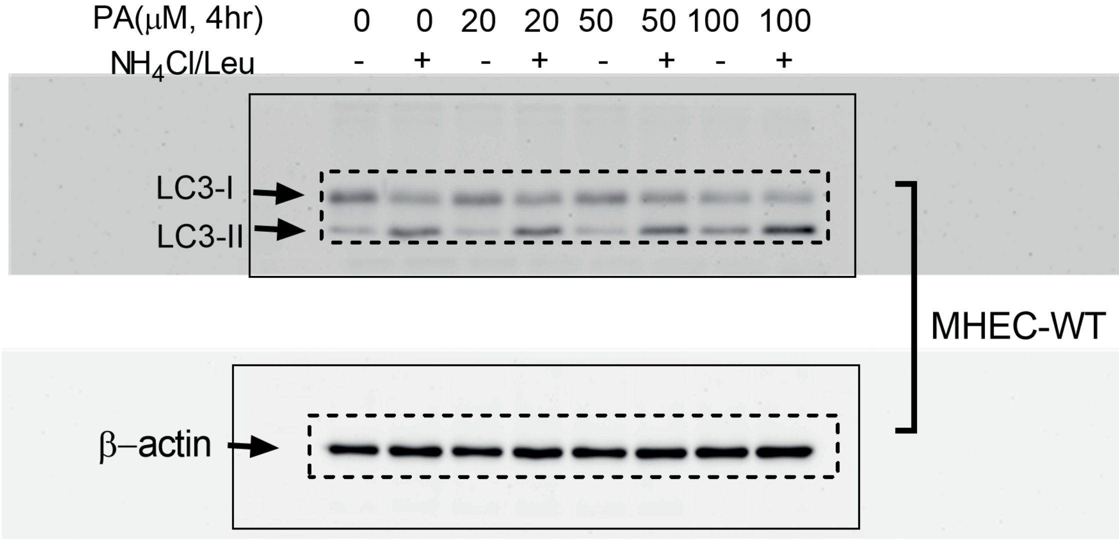

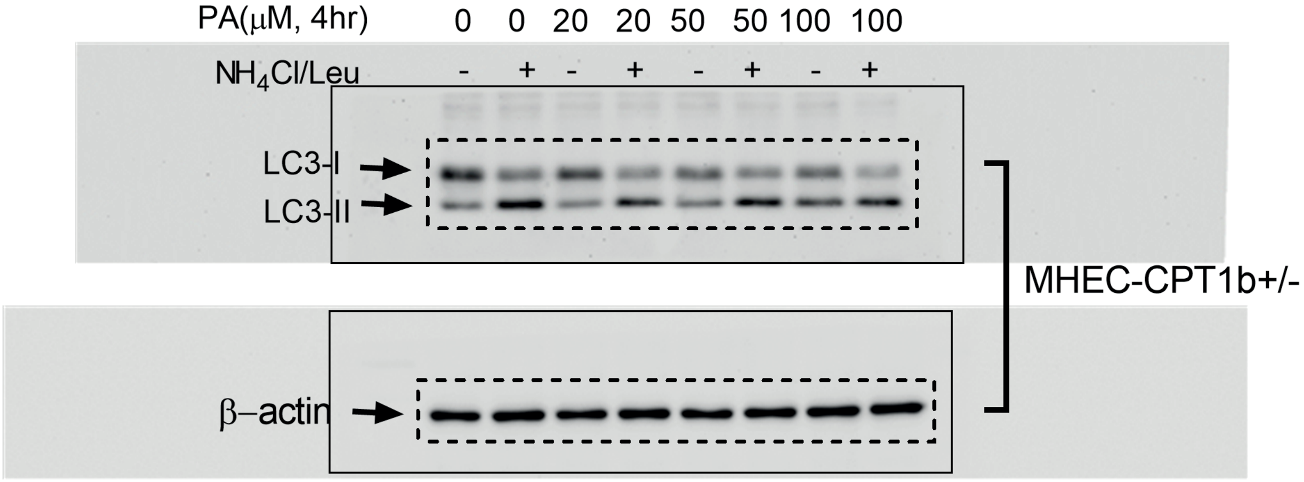

Supplemental Figure S2D. Orginal scans associated with Figure 2D.
Dottted lines are the cropped area, and the solid lines are the edge of the blots.

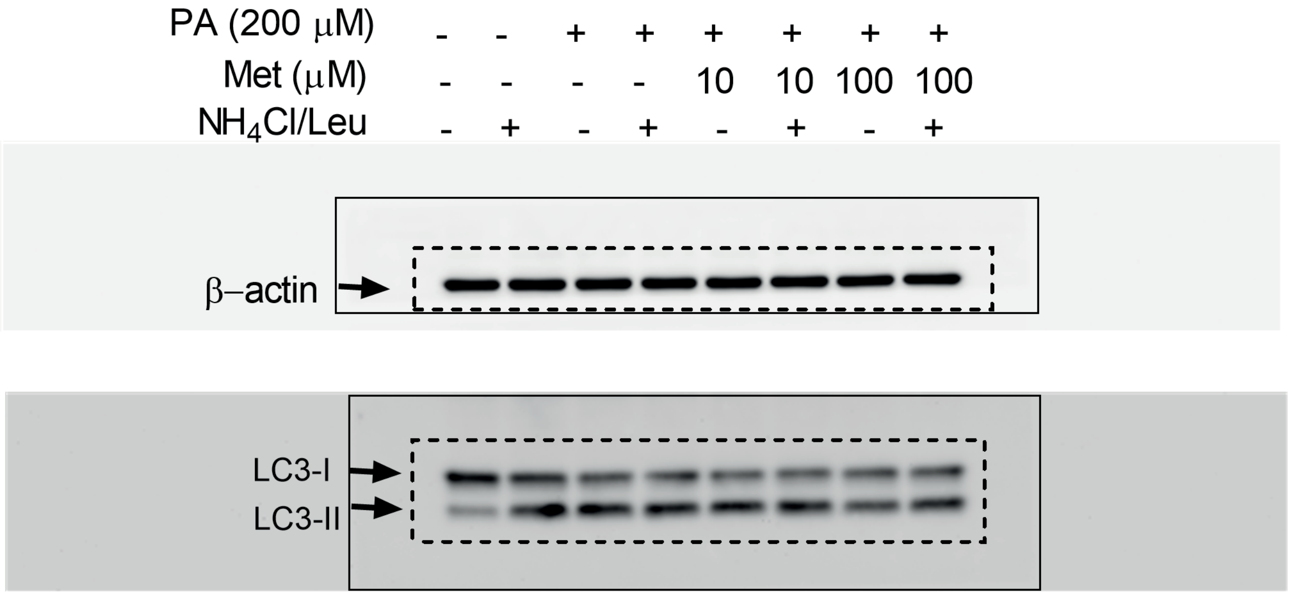

Supplemental Figure S3A. Orginal scans associated with Figure 3A.
Dottted lines are the cropped area, and the solid lines are the edge of the blots.

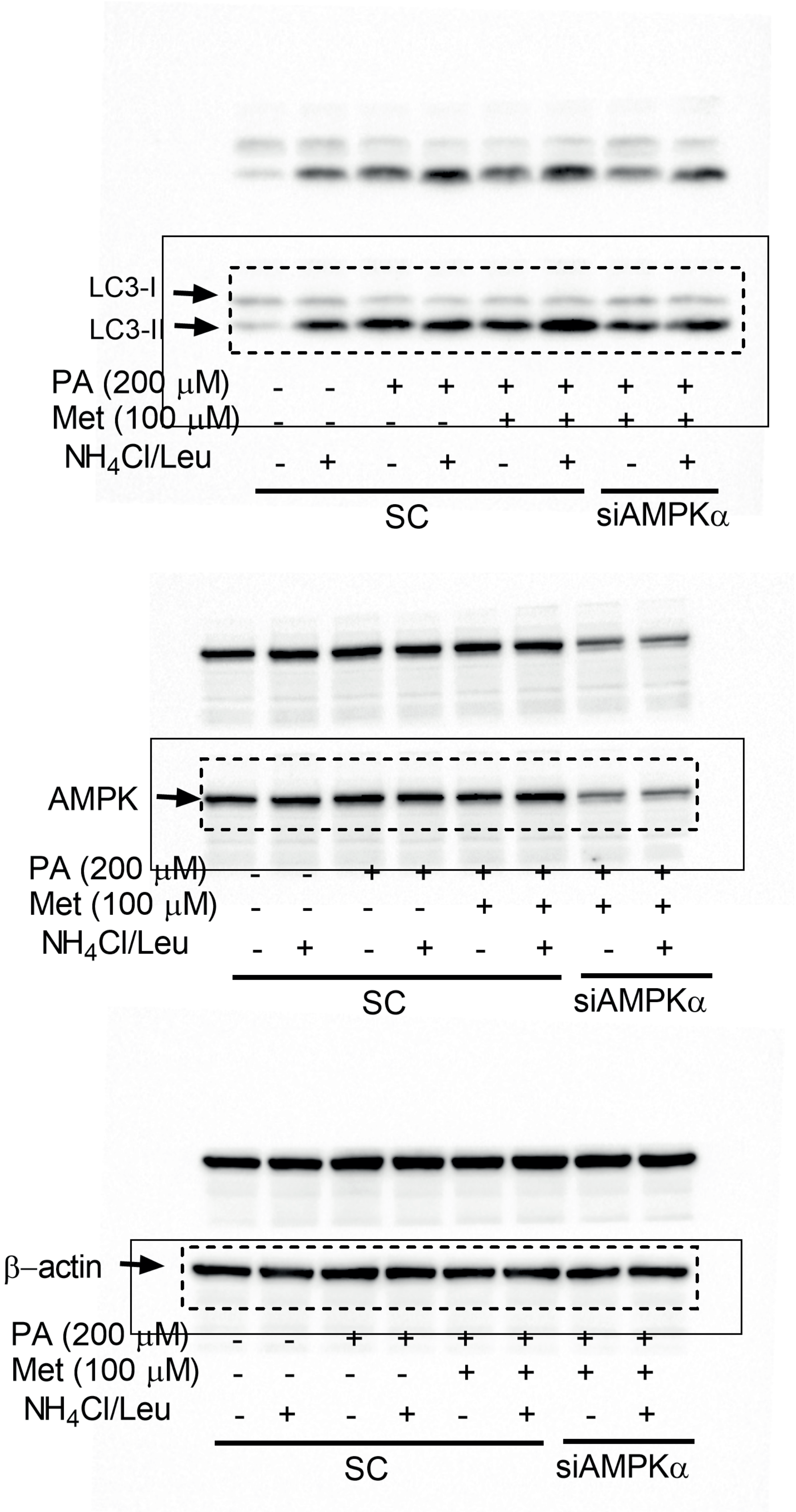

Supplemental Figure S3B. Orginal scans associated with Figure 3B. Dottted lines are the cropped area, and the solid lines are the edge of the blots.

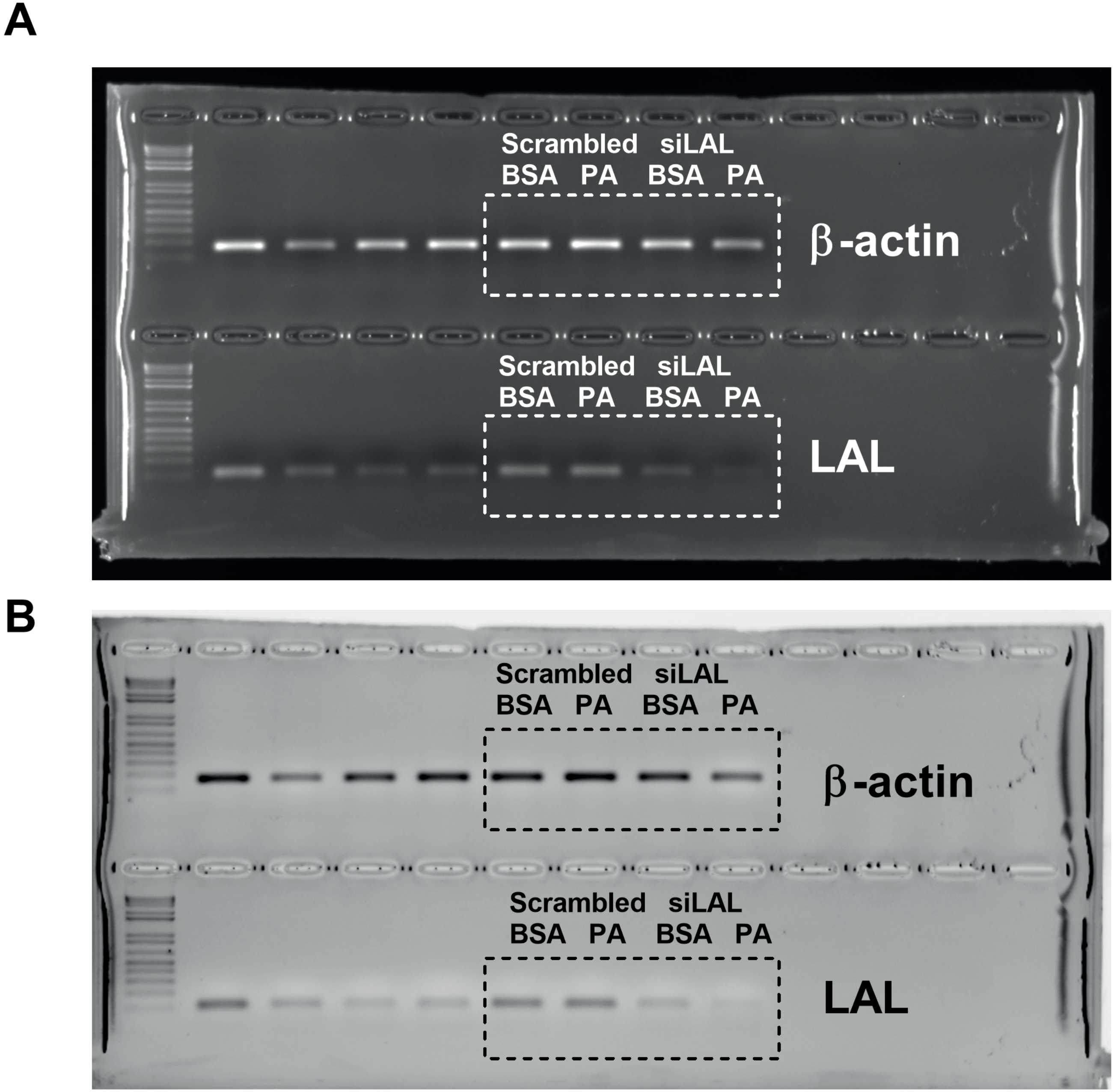

Supplemental Figure S5B. Orginal scans associated with Figure 5B
A: Agarose gel B: Inverted Image of A. Dotted lines are cropped area.
